# Examination of the Structure and Formation *Streptococcus mutans* Biofilm Induced by Glucose, Lactose, Soy Protein, and Iron

**DOI:** 10.1055/s-0043-1776121

**Published:** 2024-03-31

**Authors:** Indah Listiana Kriswandini, Hendrik Setia Budi, Fuadia Mumaiyyiah Justitia

**Affiliations:** 1 Department of Oral Biology, Faculty of Dental Medicine, Universitas Airlangga, Surabaya, Indonesia; 2 Department of Oral Biology, Dental Pharmacology, Faculty of Dental Medicine, Universitas Airlangga, Surabaya, Indonesia

**Keywords:** infectious disease, biofilm thickness, biofilm structure, chemical element, *Streptococcus mutans*

## Abstract

**Objective** *Streptococcus mutans* , the main causative agent of caries, have the ability to form biofilms on the surface of teeth. The availability of nutrients such as glucose, lactose, soy protein, and iron can influence *S. mutans* in biofilm formation. All four sources of nutrients have been shown to increase the formation of *S. mutans* biofilms. The purpose of this study was to determine the structure and thickness of *S. mutans* biofilms induced by glucose, lactose, soy protein, and iron.

**Materials and Methods** This experimental laboratory study aimed to examine the formation of biofilm structures (chemical elements) and determine the thickness of *S. mutans* biofilms induced by glucose, lactose, soy protein, and iron. The structures (chemical elements) were examined using scanning electron microscopy-energy-dispersive X-ray (SEM-EDX) analysis. Confocal laser scanning microscopy (CLSM) was used to determine the thickness of *S. mutans* biofilms with an Olympus FV1000 microscope, and the findings were analyzed using Olympus Fluoview Ver. 4.2a software.

**Results** It was established that the results of SEM-EDX examination of the structure of *S. mutans* biofilms induced by glucose had oxygen (O) as the dominant chemical element (30.24 w%); lactose reported oxygen (O) as the dominant element (29.65 w%); soy protein had carbon (C) as the dominant element (34.31 w%); and iron showed oxygen (O) as the dominant element (32.51 w%). The thickness (measured by the CLSM examination) of biofilms induced by glucose, lactose, soy protein, and iron were 17,666, 12,666, 18,000, and 15,666 nm, respectively.

**Conclusion** The structure of *S. mutans* biofilms induced by glucose, lactose, and iron contain the following elements in amounts from the highest to lowest: O, C, N, P, and S; the biofilm produced by *S. mutans* induced by soy protein in amounts from the highest to lowest comprised the elements: C, O, N, S, and P. The *S. mutans* biofilms induced by soy protein had the maximum thickness, followed by those induced by glucose, iron, and lactose.

## Introduction

Biofilms have a very heterogeneous composition: microbial cell microcolonies, polysaccharides, proteins, deoxyribonucleic acid (DNA), ribonucleic acid, and water. 1 Polysaccharides can be intracellular polysaccharides and extracellular polysaccharides (EPS). EPS are mainly *glucans* synthesized by glucosyltransferase-producing bacteria (GTFs). EPS contribute to stabilizing the biofilm matrix, supporting biochemical and physiological changes in it, increasing its porosity, improving the attachment of bacterial microcolonies to the surface, and improving the acidogenicity of the biofilm matrix. Biofilms have a main structure consisting of two main components: *water channels* that function as nutrient transport and dense cell areas that do not have pores. 2 3 4 Biofilms have various structures (three-dimensional [3D] structures) and biochemical compositions influenced by microbial species and environmental conditions. 5 6

One of the main causative agents of caries in human teeth is the *Streptococcus mutans* bacteria. *S. mutans* has the ability to form biofilms on tooth surfaces. The ability of *S. mutans* bacteria to form biofilms is influenced by their capacity to produce three types of glucosyltransferases or GTFs (GTFB, GTFC, GTFD), several glucan-binding proteins or Gbps (GbpA, GbpB, GbpC), and antigen c protein (PAc), which correlates to the virulence and formation of *S. mutans* biofilms. 7 Research demonstrates that bacterial cells in biofilms are more resistant to antibiotics and acids compared to bacterial cells found elsewhere. It is fundamental that biofilm formation is an essential requirement for bacteria to survive dynamic environmental changes. 8

The occurrence of dental caries largely depends on the role of biofilms. Factors such as the conditions of the oral environment, the availability of nutrients, and the presence of certain organic and inorganic molecules, act as signals in the formation of biofilms. The type of nutrient plays an important role in the formation of biofilms. Several studies have established that nutrients having a significant role in the formation of biofilms include monosaccharide carbohydrates and disaccharides (glucose and lactose), soy protein, and iron.

Carbohydrates have also been recognized as a major factor in biochemical and physiological changes in dental biofilms. In addition, long-term carbohydrate consumption also increases the growth of the number of *S. mutans* . 3 Carbohydrates are also known to contribute to the formation of biofilm structures. Glucose is a monosaccharide that can be directly metabolized by microorganisms, while lactose is an oligosaccharide belonging to the disaccharide group. Both are derivatives of carbohydrates and have been shown to increase the formation of *S. mutans* biofilms. Glucose for *S. mutans* acts as a substrate for intracellular and extracellular polysaccharide synthesis and increases acid production. The presence of glucose may assist *S. mutans* in producing karyogenic biofilms. 9 10 11 A study 12 reported that adding lactose to the growth medium significantly stimulated the formation of biofilms from *S. mutans* .

Soy protein has low amino acid sulfur content, but it is equivalent to the sulfur content in animal protein. Soybeans have a protein content of 40%. Soy protein has many benefits for humans, so many foods and beverages are produced using soy ingredients, including milk. 13 Soy milk has high protein content; hence, it can be used as a substitute for protein sources derived from cow's milk. Based on references, protein levels (soy milk) range from 1.16 to 2.04%. 14 According to the results of a study, 15 the levels of acid produced by *S. mutans* at pH 6.5 in all soy drinks were five to six times higher than in cow's milk samples. It may be indicated that soy drinks have a higher karyogenic potential. Iron has been shown to increase biofilm formation by several microorganisms, such as *S. mutans* , *Pseudomonas aeruginosa* , and *Staphylococcus aureus* . 16

This study aims to determine the structure (chemical elements) and thickness of *S. mutans* bacteria biofilms induced using glucose, lactose, iron, and soy protein. It is expected that materials that can play a role in the formation of bacterial biofilms can accelerate the formation of dental caries.

## Materials and Methods

### Preparation of Inoculum *S. mutans*

This study is an observational analytical study with a laboratory approach in the form of sample testing. The study was conducted between May and September 2019. The research sample was a stock of *S. mutans* bacteria (ATCC 51656) obtained from the Research Center Institute of the Faculty of Dentistry, Universitas Airlangga, Surabaya, Indonesia. S. *mutans* bacteria were cultured in tubes containing Brain Heart Infusion Broth (BHIB) medium and incubated for 2 × 24 hours in an anaerobic atmosphere with a temperature of 37°C. After that, the culture was standardized using the McFarland 5 Standard (1 McFarland = 3 × 108 CFU/mL).

### Induction of *S. mutans* to Produce Biofilms

The five groups of S. mutans was induced by 5% glucose, 5% lactose, 5% soy protein, and 2% iron, respectively. For the *S. mutans* biofilms to grow optimally, inducer concentration was selected based on previous research; hence, the biofilm protein was induced by 5% glucose, lactose, and soy protein and 2% iron inducer according to a U.S. patent. 17 After inducing, all media were anaerobically incubated for 1 × 24 hours at 37°C (using the Oxoid Anaerobic Jar system).

Each induced bacterial suspension produced a biofilm, which was distilled with phosphate-buffered saline (PBS) pH 7.4 and centrifuged at a speed of 3,000 revolutions per minute to remove the planktonic phase of the biofilm.

### Preparation of Scanning Electron Microscopy-Energy Dispersive X-Ray on *S. mutans* Biofilm

The biofilm was then fixed using 2% glutaraldehyde for 2 to 3 hours at 4°C. After that, repeated rinsing was performed with PBS and stratified alcohol dehydration. The sample was then freeze-dried using critical point drying and placed in a stand, and the biofilm was coated with a gold coating material (Au). Subsequently, the biofilm was read using the scanning electron microscopy-energy dispersive X-ray (SEM-EDX) technique. It is expected that the samples given the treatment will produce different *S. mutans* biofilm structures and thicknesses. For each biofilm on the stand, five random images were taken of the location during SEM examination with a magnification of 3,500 × , and they were further examined with EDX using the APEXTM analysis software from EDAX (SEM-EDX).

### Confocal Laser Scanning Microscopy on *S. mutans* Biofilms

The bacterial culture suspension (800 μL) was transferred to four microplates of the well, and the biofilm was planted on the surface of the round cover clip. Each induction inducer, 5% glucose, 5% lactose 40 μL, 5% TSB (Tripticase Soy Broth) 400 μL, and 2% FeCl _3_ 2 μL were incubated for 24 hours. The BHIB bacterial culture was removed and rinsed thrice with PBS and allowed to stand for 10 minutes. The PBS was changed to remove the planktonic phase, and the biofilm was fixed for 20 minutes using 4% paraformaldehyde 2 μL in the well. The fixation fluid was removed and rinsed again with PBS. The biofilm was stained with concanavalin A (ConA)-fluorescein isothiocyanate (FITC) solution, and incubated for 15 minutes, then removed and continued with a second stain using propidium iodide (PI) for 15 minutes, discarding the PI solution. The samples were transferred to a glass cover and each sample was followed by random sampling from three locations on the confocal laser scanning microscopy (CLSM) examination. The data obtained were analyzed using Olympus Fluoview Ver. 4.2a program.

## Result

### Biofilm Imaging *of S. mutans* by SEM-EDX

**Fig. 1:**
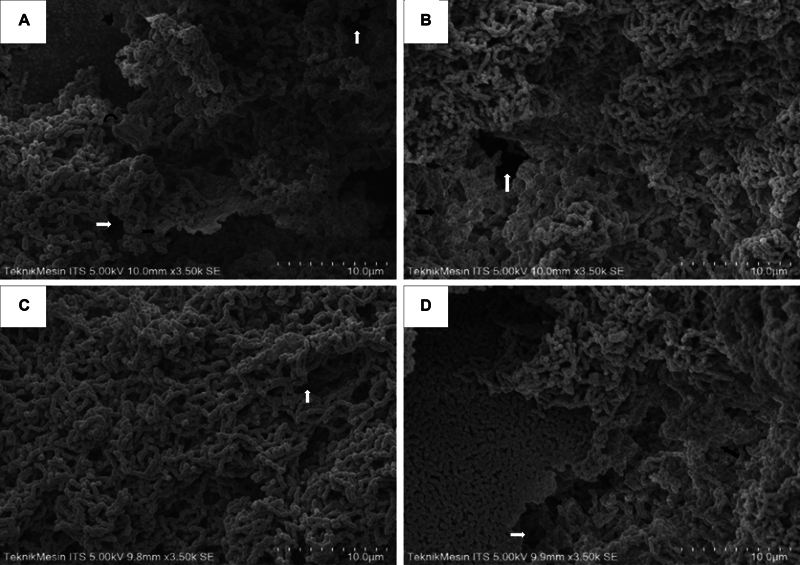
Scanning electron microscopy (SEM) results showing *S. mutans* biofilm after being induced by ( **A** ) glucose; ( **B** ) lactose; ( **C** ) soy protein; and ( **D** ) iron, with a magnification of 3,500 × . There is a black gap called a drain (white arrow), and an extracellular polysaccharides (EPS) layer (black arrow) is obtained. *S .mutans* biofilms induced by glucose, lactose, and iron are more porous. In contrast, those induced by soy proteins are denser, with *S. mutans* cell aggregation forming EPS-coated cocci in almost the entire field of view.

Observations of *S. mutans* biofilms with SEM are presented in Fig. 1 . SEM images depict the density of S. *mutans* biofilms induced with glucose 5% (A), lactose 5% (B), soybeans (using Trypticase soy broth [TSB] medium) (C), and FeCl3 2% (D). The formations of *S. mutans* biofilms in Fig. 1A, B , and D present the same density, except in Fig. 1C , which demonstrates that soybean-induced S. *mutans* biofilms (grown on TSB) have a higher density. Observations continued to examine the structure of biofilms (chemical element content) using the EDX software (APEXTM analysis software) from EDAX. The calculation of the average chemical element (w%) in *S. mutans* biofilm is recorded in Table 1 . The table illustrates the different amounts of chemical elements C, N, O, P, and S in each *S. mutans* biofilm induced with different inducers.

**Table 1:** Chemical elements (w%) in the biofilm *Streptococcus mutans*

Inducers	Chemical elements (w%)				
	C	N	O	P	S
Glucose	30.24	21.43	34.05	9.53	4.74
Lactose	29.28	18.80	29.65	16.43	5.82
Soy protein	34.31	16.48	34.16	6.97	8.07
Iron	30.78	21.49	32.51	11.20	4.01

### Biofilm Imaging *of S. mutans* by CLSM

**Fig. 2:**
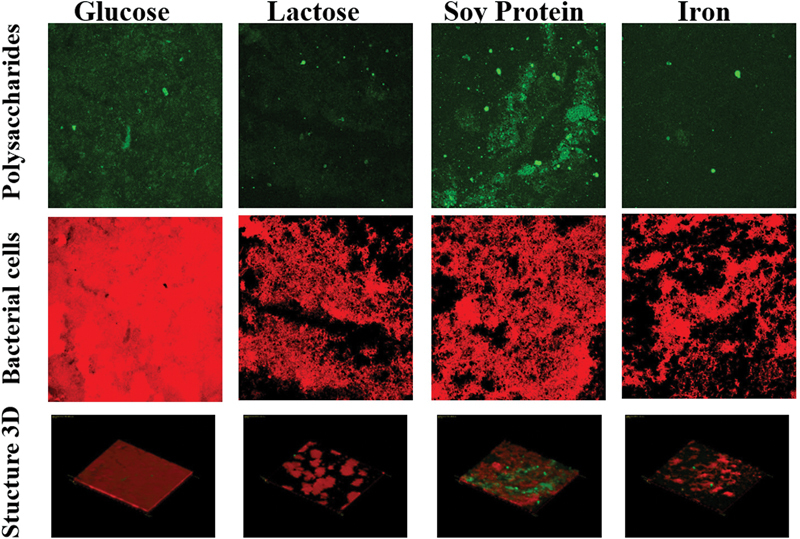
Confocal laser scanning microscopy (CLSM) observation at 400× magnification. Biofilm staining of *S. mutans* using concanavalin A-fluorescein isothiocyanate (ConA-FITC) (green) and propidium iodide (PI) (red). ConA-FITC staining polysaccharide (extracellular polysaccharides [EPS] representative) and PI staining bacterial cell *S. mutans.*

**Fig. 3:**
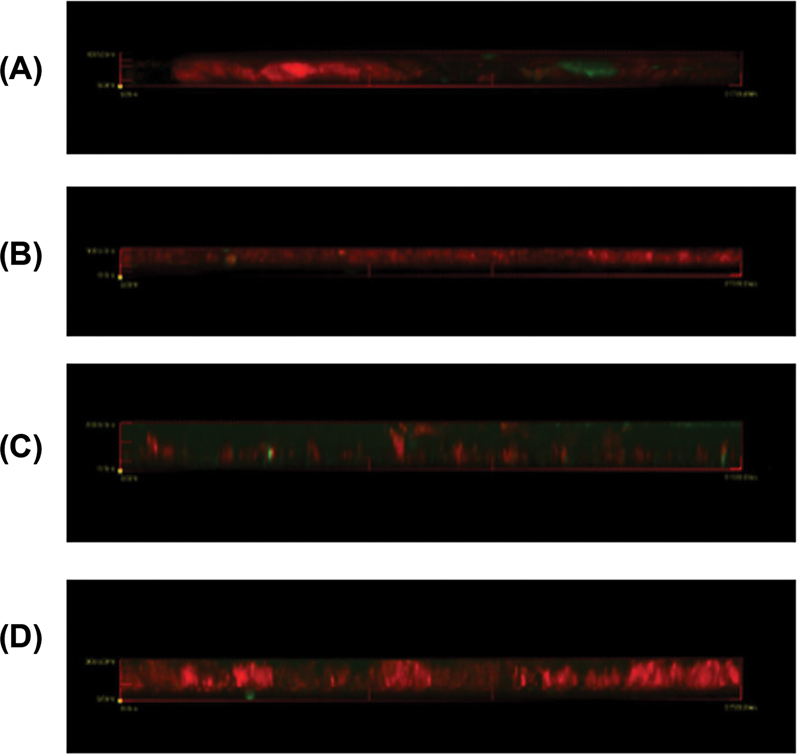
Biofilm thickness ( **A** ) glucose; ( **B** ) lactose; ( **C** ) soy protein; and ( **D** ) iron.

Observations of *S. mutans* biofilms with CLSM are presented in Figs. 2 and 3 . The quantitative results are the calculation of the average fluorescence intensity of ConA-FITC and PI, as well as the average thickness of *S. mutans* biofilms shown in Table 2 .

**Table 2:** Fluorescence intensity of ConA-FITC, PI, and thickness of biofilm *Streptococcus mutans*

Arbitrary and thickness	Inducer			
	Glucose	Lactose	Soy protein	Iron
ConA-FITC (arbitrary unit)	867.19	386.12	927.24	596.87
PI (arbitrary unit)	2,471.23	1,407.38	1,832.18	1,369.32
Thickness (nm)	17,666	12,333	18,000	15,666

Abbreviations: ConA-FITC, concanavalin A-fluorescein isothiocyanate; PI, propidium iodide.

## Discussion

Polysaccharides in the biofilm matrix contribute to cohesion, water retention, and absorption of organic and inorganic compounds. Polysaccharide staining generally cannot be obtained because the chemical structure of the polysaccharide matrix varies between different bacteria. A better approach is to use fluorescently labeled lectins so that they can recognize the disaccharides or trisaccharides contained in the EPS matrix. ConA-FITC is used as an EPS material stain matrix. Another component of biofilms, environmental DNA, can be analyzed using DNA-binding fluorescent stains such as PI. 18 19 In this study, fluorescent staining of *S. mutans* biofilm was conducted using lectins labeled ConA-FITC or FITC, which bind polysaccharides so that they can be used to detect EPS matrix and PI dye to detect bacterial cells. Both staining results on 3D biofilm structure images and *S. mutans* biofilm thickness were obtained through Olympus FV1000 and Olympus Fluoview Ver. 4.2a type CLSM software. Qualitatively, Fig. 3 shows the difference in thickness of *S. mutans* per inducer. This is possible because all four inducers have their own pathways that can induce biofilms.

In this study, it was observed that biofilms induced by soy protein were denser with the aggregation of *S. mutans* bacteria coated with EPS to meet one field of view. In glucose, lactose, and iron, porous combinations were observed between cell aggregation, EPS, and aqueducts. Porous biofilms tend to have greater substrate diffusion potential, thereby increasing metabolism and acid production by *S. mutans* . 20 After examining the S. *mutans* biofilms using SEM and then analyzing the structure of biofilms that have chemical elements using EDX (the APEXTM analysis software from EDAX), it has been determined that each biofilm sample has different average amounts of chemical elements: C, N, O, P, and S. Based on the results of the study, differences in amounts of C, N, O, P, and S were obtained for each inducer, elaborated further in the discussion.

The glucose-induced structure of *S. mutans* has an average chemical component of C (30.24 w%), N (21.43 w%), O (34.05 w%), P (9.53 w%), and S (4.74 w%). Glucose is the best source of C, which plays a role in metabolic activity and is the main energy source in bacterial growth. As is the case with carbon sources (C), bacteria need other sources such as N, P, and S for metabolic processes. 21 This allows the O contained to become dominant. The *S. mutans* glucose-induced biofilm has a thickness of 17.666 nm with an average fluorescence intensity of PI produced by 2.471 arbitrary units (au) and an average fluorescence intensity of ConA-FITC of 867 au. Glucose increases the activity of GTFB, which acts as an interaction between *S. mutants* , responsible for the formation of microcolonies that form biofilm structures and synthesize insoluble glucans. 22 A study 23 also concluded that glucose had the greatest effect on the expression of genes associated with *S. mutans* biofilms.

*S. mutans* induced by lactose have an average chemical element of C (29.28 w%), N (18.80 w%), O (29.65 w%), P (16.43 w%), and S (5.82 w%). Lactose and glucose are carbon sources for bacteria. However, bacteria will first use glucose as the main carbon source if present; otherwise, the bacteria will switch to lactose. 24 The lactose-induced *S. mutans* biofilm has a thickness of 12.333 nm with an average fluorescence intensity of PI produced by 1.407 au and an average fluorescence intensity of ConA-FITC of 386 au. In this study, the *S. mutans* biofilms induced by lactose were not as many as biofilms induced with other. Based on research, 12 lactose has specific effects that can increase the formation of biofilms, and the EPS produced is different from other carbohydrates (sucrose). Lactose is carried to cells through the phosphotransferase system and then metabolized by *S. mutans* , which produces lactose-6-P, this molecule being a signal to regulate genes related to biofilm formation. These genes are similar to the brpA, gtf, and ftf. Lactose metabolism by *S. mutans* is more related to acid and energy production but not as a substrate that produces dextran for EPS synthesis. 12 25 26

The structure of *S. mutans* biofilms induced by soy protein has an average carbon chemical element (C) of 34.31 w%, N (16.48 w%), O (34.16 w%), P (6.97 w%), and S (8.07 w%). *S. mutans* biofilms induced by soy protein have a thickness of 18,000 nm with an average fluorescence intensity of PI produced by 1,832 au and a ConA-FITC fluorescence intensity of 927 au. In the growth of *S. mutans* bacteria, several combinations of sources are needed, namely C, N, S, and P, inorganic minerals, nucleotides, vitamins, and cofactors. 27 Soy protein contains 18 types of amino acids: 9 types of essential amino acids and 9 types of nonessential amino acids. Methionine is one of the essential amino acids contained in soy protein. 28 In the results, 27 cysteine and cystine elimination did not significantly reduce the growth of *S. mutans* due to indications that *S. mutans* catalyzed another sulfur source, methionine.

The iron-induced biofilm structure of *S. mutans* has an average carbon chemical element C of 30.78 w%, N (21.49 w%), O (32.51 w%), P (11.20 w%), and S (4.01 w%). The thickness of the iron-induced *S.* *mutans* biofilm was 15.666 nm, with the mean fluorescence intensity of PI produced by 1.369 au and the average fluorescence intensity of ConA-FITC was 596 au. Iron is an essential element for many microorganisms as a major component in electron transfer and enzyme reactions and acts as a signaling molecule during biofilm formation. 16 *S. mutans* bacteria produce siderophores in order to absorb iron in the form of Fe3 + , which is then converted into Fe2+ so that it can be used for electron transfer. In several studies, it has been shown that iron has a considerable effect on the formation of biofilms and as a crosslinking agent that induces covalent bonds between polymers, thereby stabilizing the biofilm matrix. 29 30

The formation of the chemical composition contained in the biofilm is influenced by the balance between the extracellular matrix that acts as a physical barrier affecting the diffusion process of substances entering or leaving the biofilm and substances metabolized from the microbes. Chemical elements such as C, N, O, P, and S are some of the basic elements of proteins. There are many important protein forms in the biofilms of *S. mutans* , ranging from crosslinked proteins to nucleic acids, proteins that bind to polysaccharides, also called glycoproteins, glucosyltransferases, fructosyltransferases, dextranases, etc. 5 31 32

Biofilm thickness has an important role when bacteria evade the host's immune response and in resistance to antibacterial agents. 8 According to research, 33 the rate of biofilm formation is in line with certain nutrients, so it reflects the composition and thickness of different biofilms. This is evidenced in this study as it obtained different thicknesses depending on the induction. It has been established by research studies 34 that thickness has a significant impact on the ecological function of biofilms. The bacteria that thrive in the biofilm are protected from various environmental conditions such as the action of antibiotics, host immune response, and mechanical leaching, so that ability will increase with increasing thickness of the biofilm.

It can be concluded that the structure of *S. mutans* biofilms induced by glucose, lactose, and iron has the highest average chemical elements O, C, N, P, and S. In contrast, those induced by soy protein have the elements C, O, N, S, and P. The thickest *S. mutans* biofilms are induced by soy proteins, followed by glucose, iron, and lactose.

The urgency of this research is to determine percentage of chemical structure (chemical elements) contained in *S. mutans* biofilms induced with various nutrients mentioned above will be able to know the survival of the biofilm-forming microbes ( *S. mutans* ) so that this will affect its virulence. The limitation of this study is that more advanced devices are needed to detect microbial biofilm components.

## Conclusion

Analyses of chemical elements with SEM-EDX yield different elements in the entire sample and cannot distinguish polysaccharide compounds, proteins, fats, or extracellular DNA formed from these elements. Further examination of the binding of chemical elements in *S. mutans* biofilms is required to predict the chemical constituent proteins formed in biofilms after being induced by glucose, lactose, soy protein, and iron.
